# Corrosion Characterization at Surface and Subsurface of Iron-Based Buried Water Pipelines

**DOI:** 10.3390/ma14195877

**Published:** 2021-10-07

**Authors:** Dessalegn Ahmed Yeshanew, Moera Gutu Jiru, Gulam Mohammed Sayeed Ahmed, Irfan Anjum Badruddin, Manzoore Elahi M. Soudagar, Sarfaraz Kamangar, Mesay Alemu Tolcha

**Affiliations:** 1 Department of Mechanical Design and Manufacturing Engineering, ADAMA Science and Technology University, Adama 1888, Ethiopia; desalegn4719@gmail.com; 2 Program of Mechanical Design and Manufacturing Engineering, ADAMA Science and Technology University, Adama 1888, Ethiopia; 3 Program of Mechanical Design and Manufacturing Engineering, School of Mechanical, Chemical and Materials Engineering (So-M-C-M-E), ADAMA Science and Technology University, Adama 1888, Ethiopia; drgmsa786@gmail.com; 4 Mechanical Engineering Department, College of Engineering, King Khalid University, Abha 61421, Saudi Arabia; magami.irfan@gmail.com (I.A.B.); sarfaraz.kamangar@gmail.com (S.K.); 5 Department of Mechanical Engineering, School of Technology, Glocal University, Delhi-Yamunotri Marg, SH-47, Mirzapur Pole, Saharanpur District, Uttar Pradesh 247121, India; me.soudagar@gmail.com; 6 Faculty of Mechanical Engineering, Jimma Institute of Technology, Jimma University, Jimma 378, Ethiopia; alemu170@yahoo.com

**Keywords:** corrosion damage, iron pipes, surface characterization, corrosion mechanisms, image processing

## Abstract

Water pipe surface deterioration is the result of continuous electrochemical reactions attacking the surface due to the interaction of the pipe surface with environments through the time function. The study presents corrosion characterization at the surface and sub-surface of damaged ductile iron pipe (DIP) and galvanized steel (GS) pipes which served for more than 40 and 20 years, respectively. The samples were obtained from Addis Ababa city water distribution system for the analysis of corrosion morphology patterns at different surface layers. Mountains 8.2 surface analysis software was utilized based on the ISO 25178-2 watershed segmentation method to investigate corrosion features of damaged pipe surface and to evaluate maximum pit depth, area, and volume in-situ condition. Based on the analysis maximum values of pit depth, area and volume were 380 μ m, 4000 μm^2^, and 200,000 μm^3^, respectively, after 25% loss of the original 8 mm thickness of DIP. Similarly, the pit depth of the GS pipe was 390 μm whereas the maximum pit area and volume are 4000 μm^2^ and 16,000 μm^3^, respectively. In addition, characterizations of new pipes were evaluated to study microstructures by using an optical microscope (OM), and a scanning electron microscope (SEM) was used to analyze corrosion morphologies. Based on the SEM analysis, cracks were observed at the sub-surface layer of the pipes. The results show that uniform corrosion attacked the external pipe surface whereas pitting corrosion damaged the subsurface of pipes. The output of this study will be utilized by water suppliers and industries to investigate corrosion phenomena at any damage stage.

## 1. Introduction

In the designing and manufacturing process, iron-based products are favorable for making industrial products such as machine parts and pipes with the required aesthetic and functional properties. On the other hand, corrosion is a physically conditioned phenomenon of undesired results that deteriorate the integrity of iron materials which leads to damaged manufacturing products through time process. The issue of corrosion is a great problem among several stakeholders including material science engineers, water pipe distribution owners, product designers, health care, industries, hospitals, economists, researchers, etc. Moreover, buried pipes always create doubt to the owners of the distribution system due to sudden and frequent corrosion damage of the pipes.

The causes of corrosion damage mechanisms of buried metallic pipes are broadly categorized as material compositions, environment, operational, and different manufacturing process phases which leads to deteriorating aesthetic and mechanical properties [1,2]. The failure of buried pipeline probability observes that either the pipes suddenly failed from giving up service or leaking of water [3]. These effects come from the gradual degradation of the material surface leading to thin pipe thickness and weight loss before the structure completely stops its function in addition to reducing water quality [4]. One of the distinct behaviors of pipe corrosion is highly complicated due to the variability of water and soil parameters that can form corrosion attacks on both surfaces. In this case, buried pipeline safety management is important to identify the risks at the early stage [5]. Though corrosion is a physically conditioned phenomenon, it is possible to control its risks to the lowest possible level by designing and manufacturing environmentally compatible products. The external pipe surfaces of buried iron pipes are primarily affected by soil corrosion due to the presence of minerals and soil moisture acting as an electrolyte [6]. Additionally, localized pitting corrosion is a major problem that damages water pipe structures and leads to maintenance and replacement costs. Similarly, internal pipe wall corrosion formed rough irregular surfaces due to the function of viscosity, pipe structure, the velocity of water, and stagnant flow condition [7,8]. The wish of water supply owners is to supply drinking water to the consumers without losing the quality of water. However, water quality characteristics can be influenced by the interaction of water and pipe materials which leads to the creation of internal corrosion, biofilm, and leaching [9]. In the electrochemical process, iron is oxidized at the anode and released into the bulk water in the form of an ion from corrosion damaged of the pipe surface. 

Several kinds of research have been used image processing to characterize corrosion behaviors and to interpret the damage situations such as pit depth and volume [10,11] at any corrosion stage. Water pipe corrosion is categorized as electrolytic corrosion forming iron-hydroxide corrosion products and galvanic corrosion [12]. Other forms of corrosion can attack water pipe surfaces including:

- Erosion corrosion which is caused by water flow and high service load. It forms a rough surface on the pipe wall due to the different degrees of corrosion damaged surface. The corrosion products of erosion–corrosion depend on the type of compound found on the surface of the pipe.
- Uniform corrosion that formed when the pipe thickness is reduced uniformly around the buried pipe. Such type of corrosion damage is the result of a corrosion product known as magnetite; Fe_3_O_4_.
- Pitting corrosion is a localized corrosion attack forming a small hole or cavity in the metal surface. The corrosion product of pitting resulted from the interaction of iron, oxygen, and moisture or water.
- Stress corrosion cracking (SCC) is a form of corrosion caused by the internal water pressure and soil load. As the pressure exerts on the pipe surface, the circumferential and longitudinal stresses can cause cracks and develop corrosion with the combined action of stresses and the exposure to the corrosion conditions.
- Galvanic corrosion is formed between the coating material and the outer metallic surface. It can also occur in the dissimilar metallic alloying elements due to the difference in electrode potentials between the two materials.

Corrosion damage of water pipes starts from both internal and external surfaces and continues towards the subsurface depending on the exposure time and environmental factors. The formation of external corrosion scales on the surface of buried water pipes is caused by electrochemical reactions between pipe and soil materials [13]. Characterization of corrosion scales is the best technique to determine surface deterioration and corrosion morphology. It also helps to identify the behavior of corrosion products and to apply corrosion control strategies [2]. As the outer surface depends on coating type and materials, the subsurface relies on the design of the product, the proportionality of chemical compositions, and the manufacturing process especially related to the defect formation. In the electrochemical reaction, the subsurface of pipes can easily be attacked by corrosion more than the outer surface due to the rate of corrosion damage increasing as a result of pits being accumulated deposits and the rate of corrosion accelerating with the presence of chloride. 

The interaction of pipe with environmental moisture from the environment forms hydroxyl groups (–OH) and ferrous oxides on the surfaces of pipe to form corrosion products [14,15]. Surface deterioration creates the crystallographic structure of iron-hydroxide corrosion products that commonly contain β-FeOOH, γ-FeOOH, and α-FeOOH. Other forms of oxides such as Fe_3_O_4_ and Fe_2_O_3_ can be obtained through careful investigations of crystallographic structures which helps to identify corrosion mechanisms. Corrosion phenomena are complex due to a variety of parameters such as pressure which can cause unexpected pipeline failure in the chemical industry and even in the water distribution system [16]. To understand corrosion damage, it is possible to predict the process of corrosion from the data recording using a statistical method to develop a mathematical model from the set of variables such as pit depth, age, operation condition, and pipe material information [17]. There are a variety of corrosion factors that influence the corrosion process on the pipe surface, including water and soil parameters in addition to stresses subjected to the pipe. 

Soil types and compositions are inherently different from place to place due to the variability of soil mineral elements [18]. The composition of soil and grain size are very important parameters for the formation of corrosion reacting with the pipe surface. For example, fine grain size is found in clay soil which can contain more water than loam and sand soil types. A soil that can contain more water is more corrosive than other soil types. The increasing order of soil moisture-holding capacity is sandy, load, and clay soil type which corresponds with increasing corrosion possibility. Several studies show the soil type relating with corrosivity level and categorized in increasing order as sand–clay–load, clay–loam, and clay soil [19,20]. The safe condition of soil type is sandy soil for the underground pipelines due to very little water content. 

Buried water pipes have been influenced by external corrosion of overlying soil and internal corrosion caused by water parameters which consequentially cause various losses including public health, cost of water treatment, and the economy at large for pipe maintenance and replacement. Among water parameters, conductivity, total dissolved solids, the deposit of trace elements, dissolved oxygen, chloride, and pH of the water are more responsible for pipeline damage. Similarly, soil corrosivity is a common problem that affects pipe service lifetime and challenges pipe manufacturers. Some soil parameters including moisture, pH, resistivity, density, soil element, and soil type are major factors that affect external pipe surface failure. 

During service time, the internal pipe surface wall gets rough, and turbulent and laminar flows develop into the bubbling of the water which indicates oxygen entrapment in the water forming erosion–corrosion that affects the corrosion of the pipe, severely reacting with water to form hydroxide and ferrous ions as shown in Equations (1) and (2).

(1) $$O_{2}+\frac{1}{2}H_{2}O\to 4{\mathrm{OH}}^{-}$$

Fe→Fe^2+^ + 2e^−^ (2)

**Figure 1:**
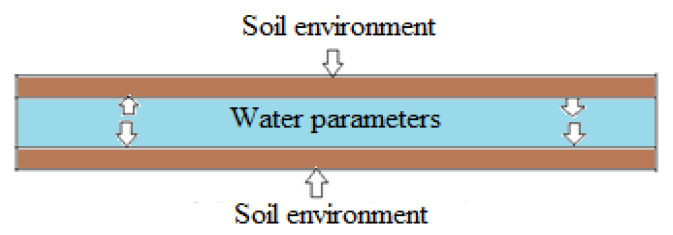
Schematic of the buried water pipe and its environments.

Corrosion can be analyzed using imaging instruments to characterize the surface and crystals of corrosion products by using scanning electron spectroscopy (SEM) to assess corrosion morphologies and X-ray diffraction spectroscopy (XRD) for studying corrosion products [21,22,23]. These instruments have the potential to provide internal and external detailed information on corrosion products by identifying the composition, structure, and morphology of corrosion. Image processing is an effective corrosion characterization technique from 2D and reconstructed 3D images to evaluate the geometric features of the pit and profile of surface irregularities [24]. The corrosion behaviors of soil and water environments are responsible for the lifetime of iron pipes due to the direct contact with pipe surfaces, as shown in Figure 1.

Internal pipe corrosion can be caused by trace elements such as lead, copper, and others when deposited on the surface of pipes [25]. On the other hand, the external pipe surface is mainly damaged by soil resistivity, low value of pH, moisture content, and chloride [26,27]. The presence of soil electrical resistivity is the main indicator of soil corrosivity attacking the surface of pipes due to chemical content, and temperature [28]. Most works of literature studied the severity of pitting corrosion on the pipe lifetime, but this is not sufficient, as describing the attack of corrosion at the surface and subsurface of the pipe thickness before failure of the pipe is required. The focus of this study was to characterize corrosion behaviors at the surface and subsurface of drinking water pipes including metallographic and environmental factors to analyze corrosion morphology of underground galvanized steel (GS) and ductile iron pipe (DIP) in in situ conditions. The results of the study have many advantages for the owners of industries to evaluate corrosion failure at an early stage. Moreover, the information obtained from the study will be important for water distribution suppliers to investigate water quality relating to the rate of pipe surface corrosion damage as well as for predicting pipeline remaining lifetime and applying the right time of pipeline maintenance and replacement.

## 2. Materials and Methods

### 2.1. Materials

Corroded iron pipe samples were collected from different sites of the Addis Ababa city water distribution system, Ethiopia. The specimens were dug out and cut using a hacksaw from the bulk size and GS pipe with a 100 mm diameter. Similarly, corrosion damaged of 300 mm diameter of DIP collected from the water main for corrosion characterizing at surface and subsurface in-situ condition. The study covers both surfaces inside and outside of the tubes. This is due to pipe surface deterioration was affected by the inside and outside corrosive environments. From visual inspection, more surface damage was observed at the bottom of the pipes both from internal and external surfaces. The ages of galvanized steel pipe (GS) and ductile iron pipe (DIP) were more than 20 and 40 years, respectively

The new GS pipe has mechanical properties of tensile strength of 350 N/mm^2^, elongation of 14%, and yield strength of 207 N/mm^2^. The steel has British standard BS-1387 (1985). Similarly, the mechanical properties of DIP are tensile strength 480 N/mm^2^, elongation 18%, and yield strength 365 N/mm^2^. The designation of DIP pipe is ISO 2531 2009, (E) which was prepared for the analysis of microstructure.

**Figure 2:**
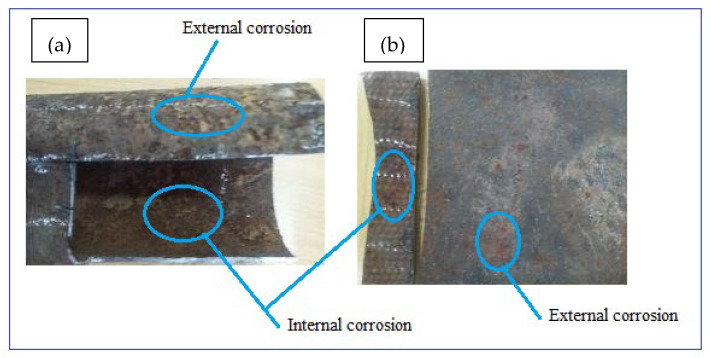
Photographs of typical corrosion damaged surfaces: (**a**) GS pipe and (**b**) DIP pipe.

Corrosion damaged samples were prepared from two possibilities of in-situ and ex-situ conditions. The ex-situ method utilized an artificially prepared mixture of chemical forming electrolytes (artificial corrosion environment) whereas the in-situ technique of sample preparation utilizes natural environmental conditions. This study preferred to investigate the samples obtained naturally damaged by the soil and water environments in the loam soil. The electrical potential of soil was 0.313 dS/m, the internal pressure of 350 KPa, flow velocity of 1.5–2.5 m/s, and the length of these parameters reaction was for more than 40 years with ductile cast iron water main. The samples were then quickly taken to the laboratory to protect further atmospheric corrosion and to remove soil particles mechanically using a plastic brush and cotton cloth before analyses. After photographing, the samples were cut into sections to evaluate the internal wall of the pipes as shown in Figure 2a,b. The surface damage thickness was compared with the original pipe thickness to determine thickness loss due to corrosion damage. Additionally, soil water and samples were taken from the same sites to study corrosion mechanisms due to environmental factors.

### 2.2. Methods

#### 2.2.1. Characterization Equipment

To achieve the objectives, the authors utilized imaging instruments including an optical microscope (OM, Huvitz HR-300 series, Huvitz, Gunpo, Korea) for analyzing internal details and scanning electron microscope (SEM) to characterize the topography of pipe materials and corrosion morphology. The laboratory analysis also focused on the microstructures of new pipes to evaluate defects from manufacturing points of view. Sample preparation of OM was carried out following standard procedures including cutting, mounting, and grinding using emery papers (grit numbers 400–2000). The samples were washed with running tap water during grinding to remove fine chips from the grounded surface before continuing to the next finer grit paper. The pipe samples were polished with a grit of 6-micrometer disc before being etched into the solution of ethanol 50 mL and 1% of HNO_3_ for 4 s to produce a mirrored surface before analysis. Additionally, six corroded samples were cut from DIP and GS pipe with an 18 × 20 mm^2^ size to study corrosion morphology by using SEM-JEOL Japan, Kyoto, Japan). The SEM images were taken several times to characterize both internal and external surfaces at the micro-level with different magnifications starting from 1 mm at a decreasing size order including (500, 200, 100, 50, and 20) μm. Below 20 μm, the image was blurred which is unable to differentiate surface details. For this study, a 100 μm scale was taken and the images were enlarged by100×–200× and 170×–220× for DIP and GS pipes, respectively. The elemental compositions of galvanized steel and ductile iron pipes were evaluated using a spark spectrometer and presented in Table 1 and Table 2, respectively. The coating thickness of the galvanized and ductile iron pipe was 55 µm and 25 µm, respectively.

Corrosion-damaged pipes were excavated, cut, and covered with a paper bag to protect atmospheric corrosion and immediately taken to the laboratory for the analysis of scanning electron microscope (SEM).

#### 2.2.2. Image Processing

The image processing (IP) was utilized to characterize corroded pipes surface topographies and pitting features considering environmental and service age. It is important to characterize the degree of deterioration caused by environmental factors during service lifetime. The IP algorithm recognizes the SEM image of the corrosion damage surface and characterizes it using the watershed segmentation method to present the results in the form of graphs and tables. The morphology of pitting behaviors was evaluated based on the concept of roughness analysis of in-situ conditions from corrosion-damaged pipe surfaces. The digital image surface analysis method was developed to determine surface morphology including pit characteristics. 

**Figure 3:**
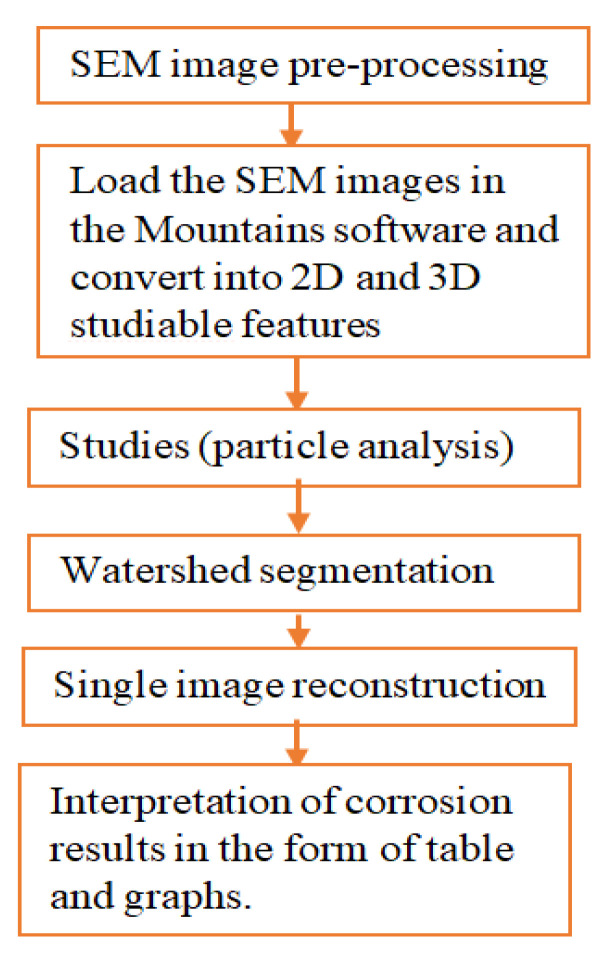
Procedures of image processing.

Using Mountains 8.2 digital image surface processing software; the SEM images were analyzed following the procedures as shown in Figure 3. The method is effective to analyze from minimum or threshold level to the maximum values of pit depth, area, diameter, and volume per particle of the selected image sample.

## 3. Result and Discussion 

### 3.1. Morphology of Corroded Pipes Surface

**Figure 4:**
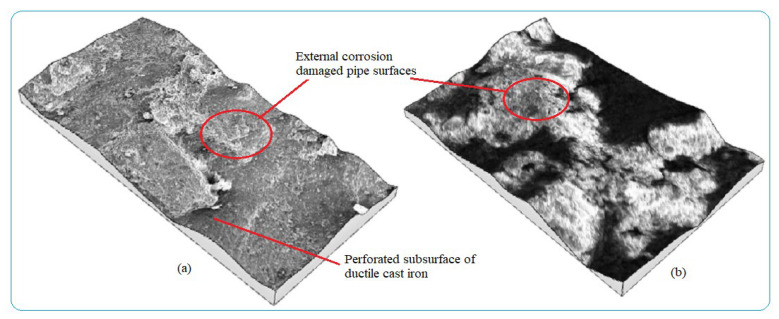
3D images of external corrosion damaged pipe surface: (**a**) Ductile iron pipe and (**b**) Galvanized steel.

Water pipes surface corrosion inspection needs early detection to evaluate the corrosion damage status of the pipes, control water contaminants, and apply maintenance activities. Analyzing the rate of pipe surface removal as a result of electrochemical reaction is useful to determine corrosion characteristics of buried pipes in a given area with a time function. Numerous factors lead to pipe failure. Some of them are easily identified by the inspection of pipeline and its surrounding environment while others require more in-depth investigation of failure factors related to pipe material, age, manufacturing method, operating conditions, soil load, soil type, etc., as the main corrosion damage mechanisms. Internal pipe wall corrosion damage was caused by different parameters but the main pipe surface damage mechanisms were grouped into two categories; viz, deposits on the wall during the stagnant condition, and localized corrosion. The forms of corrosion formed on the water pipes during the service times are shown in Figure 4 with different irregular topographies with pitting and uniform corrosions from external pipes surfaces. The entire surfaces of the pipes were damaged with non-uniformly corrosion products due to environmental corrosion variability. The features of corrosion-damaged surface morphology including pit shape, size, and depth are different depending on the behaviors of corrosion failure mechanisms. 

Pitting was formed when the passive layer locally breaks which easily attacked the pipe surface. The process of passive layer breakage was assumed as a galvanic cell that develops an anodic site where the pipe corrodes and the undamaged area acts as cathodic protection. Pipe surface corrosion damage develops at the anode due to the loss of metallic ions and a reduction reaction is formed at the cathode which consumes electrons in the process of electrochemical reaction. As the pit depth increased, the pit acts as a pocket to hold moisture, chloride, and mineral ions able to form acidic or salty depending on the content soil parameters which accelerates corrosion rate. Corrosion damage of underground ferrous pipes begins at the outer surface and then continues to the subsurface according to the soil environmental context. Studying the chemical interaction of soil–pipe surface was crucial to identify soil behavior, applying maintenance and replacement, and predicting pipes’ operational service time. Soil moisture attacked the pipe surface by forming Fe(OH)_2_ thin layer corrosion product and through time process which decomposes into magnetite in the presence of dissolved oxygen.

3Fe(OH)_2_→Fe_3_O_4_ + 2H_2_O + H_2_ (3)

External corrosion characteristics of buried iron pipes are influenced by the soil parameters of corrosion formation through a chemical or electrochemical reaction. This is due to underground pipe corrosion damage accelerates as the amount of moisture and oxygen increased in the soil. Soil parameters like electrical conductivity and soil type have their effect on the pipe surface damage. Table 3 presents the laboratory results of soil analyses. Based on soil the test, the soil corrosivity was identified and ranged from “less corrosive to moderately corrosive” from the depth of 40–120 cm.

### 3.2. Analysis of Pipes Surface Deterioration

Ductile iron and galvanized steel pipes were mainly used for water distribution due to their mechanical advantages. However, their service life was challenged by different corrosion mechanisms such as the formation of small cracks, non-uniform coating thickness, and environmental impacts during the exposure time. Through time process, buried pipes were interacting with soil and water environments in addition to operating conditions such as water flow rate and pressure conditions. From the detailed analysis, internal and external pipe corrosion mechanisms are grouped as (1) mechanical effects such as soil load and water pressure and (2) electrochemical reaction or environmental impacts with time function. The electrochemical reaction creates different stable corrosion products such as iron oxides and iron hydroxides which are the indicators of surface deterioration. The process of pipe surface damage begins when the passive film breaks on different occasions which leads to pitting formation. As a result of this, the pits continuously propagate and penetrates the surface of the pipe at random points, and later develop uniform corrosion when colonies of pits were joined each other and the process continues till pitting termination or leakage formation. 

**Figure 5:**
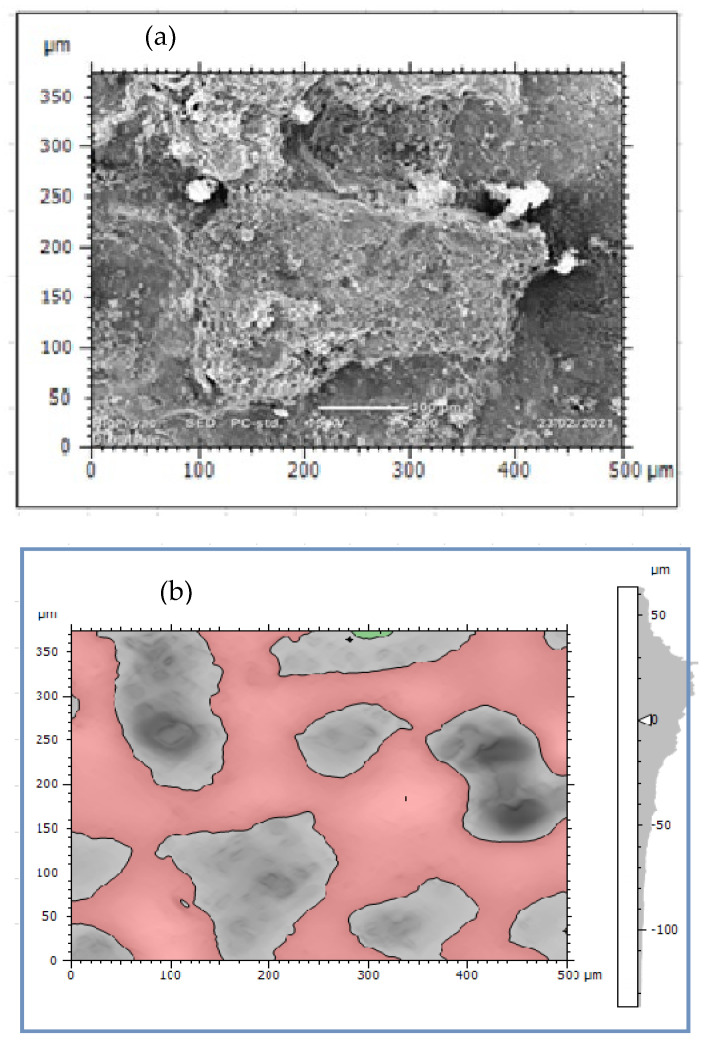
External corrosion damaged surface of DIP: (**a**) True color of SEM image and (**b**) Corrosion scale morphologies.

The rate of pipe surface deterioration accelerates with the presence of chlorine ion which dissolves iron particles at the anodic site. Others such as galvanic, uniform, and pitting corrosions are the failure mechanisms of water pipes. The formation pitting passes three stages; namely, pitting initiation, pitting growth, and pitting termination or leakage. At the initiation phase, the external surface including coating has direct contact with moisture and oxygen which can form films. The films then easily break due to electrochemical reaction and create initial pitting which damages the coating and then the external surface. The pits growth occurs at the second stage and damages the external pipe surface then it proceeds to the subsurface of the pipes due to the formation of depth. At this stage, stress corrosion cracking contributes the rate of pit corrosion to be faster and the size of pits grows easily. Depending on the variations in pit depth and width, a local colony of pits meets each other and the surface of the pipes deteriorates layer-by-layer as shown in Figure 5a. Further dissolution occurred at the anodic site and consequently, the growth of pits form pipe breakage and leakage. The cycle continued to damage the external and subsurface are easily attacked by electrochemical reactions. When the pit depth increased, it can hold more amount of moisture and oxygen which accelerated the rate of corrosion till leakage formation. This indicates the combined effect of pitting and uniform corrosions lead to surface deterioration with time function. Figure 5b presents the result of topography features of image processing obtained from corrosion morphology of DIP deteriorated surface which identified peaks and dale of the surface textures. The topography of the damaged surface was filled with watershed detection to process it for further analysis at particle level categorizing by grain boundaries. From the image analysis, it is concluded that the Mountains 8.2 surface analysis software identified the topography of corrosion failed pipe surface that the structure is deteriorated layer-by-layer creating the peaks and dale of corrosion damaged surface which indicates the process of corrosion rates.

### 3.3. Characterizations of Metallographic Microstructures

**Figure 6:**
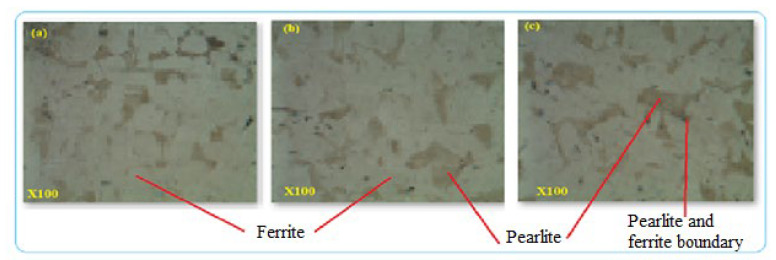
(**a**–**c**) Microstructures of GS pipe.

**Figure 7:**
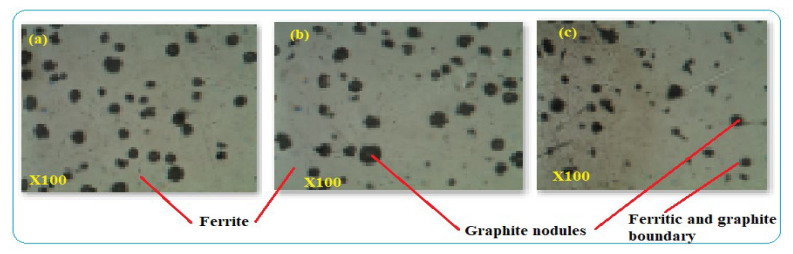
(**a**–**c**) Microstructures of DIP pipe.

The composition of alloying elements and manufacturing process were able to create defects that contribute to corrosion formation especially when there are voids and micropores in the structure. Cross-sections of metallographic structures of 4” galvanized steel water pipe were shown in Figure 6. The samples give full structural information of grain size and boundaries that were observed as uniform distribution of alloying elements. The microstructures were analyzed at a magnification of 100× under optical metallurgical microscopy and the structures were observed as pearlite with dark color and ferrite marked by white color Figure 6. The cross-sectional surface micromorphology shows that the possibility of particle deterioration formed on the surface where at the interface between ferrite and pearlite due to the two dissimilar structures acting as galvanic corrosion. The uneven distributions of alloying elements lead to microcracks at the boundaries and develop low corrosion resistance to the products which forms intergranular corrosion along the contact lines. Directional grains can create intergranular corrosion that causes the formation of microcracks in the structure. Non-uniform alloying elements and grain structures developed uneven morphologies in the internal microstructures which were formed as intergranular corrosion. The microstructure analysis was conducted from the sample of the new galvanized steel pipe cross-section, not from the top or bottom surface. The reason was to evaluate the internal details easily from the cross-sectioned part and to control the effect of zinc coating on the micrographs. Figure 7a–c are the images of optical microscope from the sections of three GS pipe samples. The images have similar internal microstructures consisting of ferrite and pearlite structures which are separated by the grain boundaries.

The typical ductile iron pipe was used for the characterization and is shown in Figure 7. The distribution and amount of alloying elements have their effect on the microstructures and defect formation. The microstructures indicated spheroidal graphite shape distributed in the DIP structure and surrounded by α-ferrite. Graphite makes the pipe have better tensile properties than other types of cast-iron pipes. The size and shape of graphite depend on the manufacturing process of the pipe. At the boundaries of graphite and pearlite or ferrite, corrosion initiation can be developed due to dissimilar structures. After 40 years, DIP pipe was deteriorating relatively at a faster rate and its safe service condition was 50% pipe thickness loss. Beyond this thickness, the rate of corrosion was faster which caused water quality reduction and consequences to pipe leakage and breakage. Figure 7a–c are the optical microscope images of the three DIP samples which were cut from the same pipe. The images comprises ferrite and graphite nodules in the microstructures of the samples. All the specimens have similar metallurgical structures and have corrosion tendency between the contacts of unlike microstructures.

### 3.4. Image Processing of Corroded Surface

**Figure 8:**
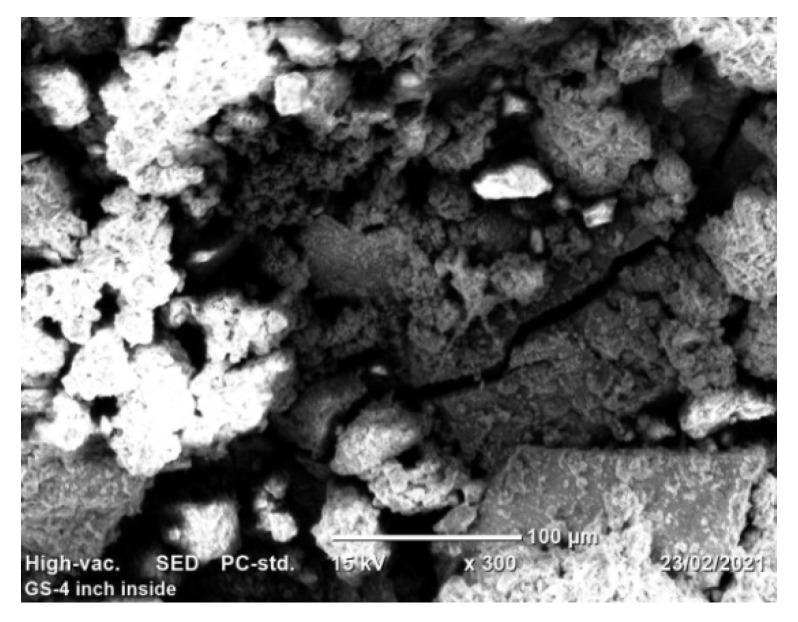
Corrosion morphology: True color of SEM image.

**Figure 9:**
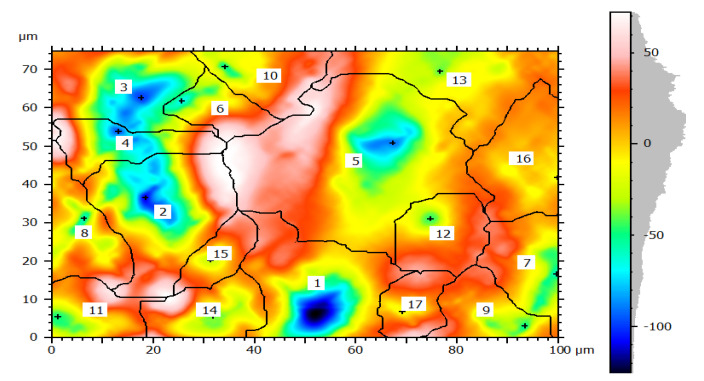
Corrosion morphology of watershed segmentation per particle.

**Figure 10:**
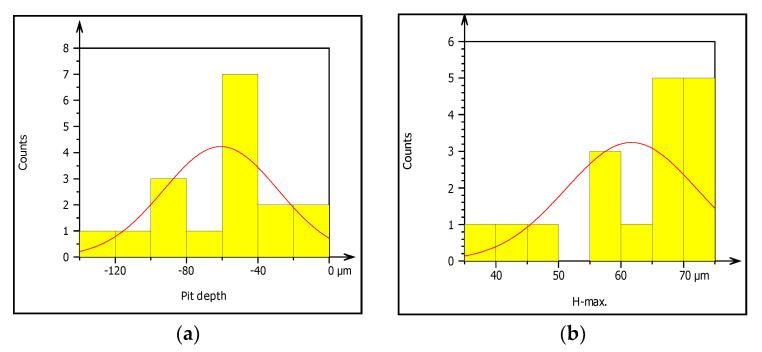

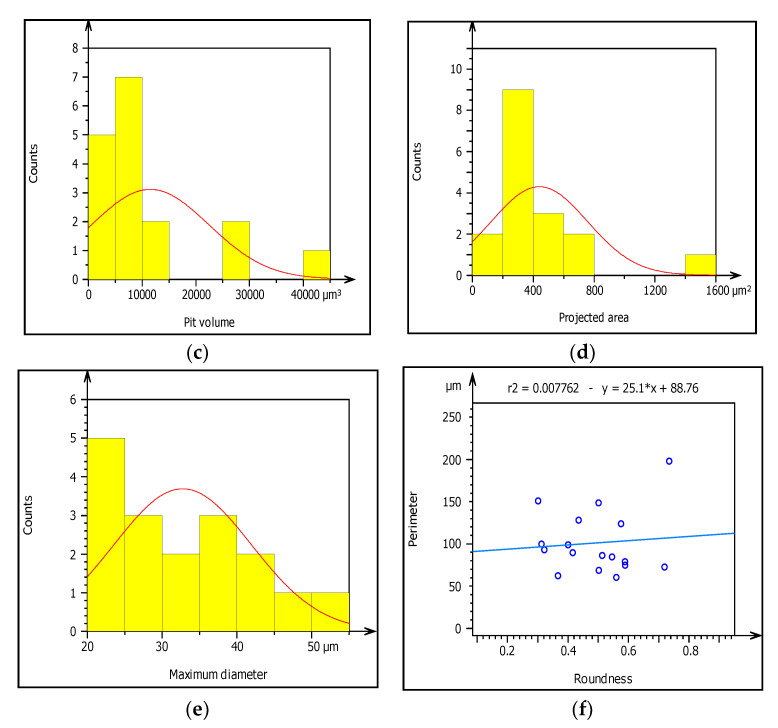
(**a**–**f**) Characterization of pitting corrosion results obtained from galvanized steel pipe.

Image processing was an alternative method of analyzing pitting corrosion and surface morphology characteristics. Mountains 8.2 software was applied for analyzing corroded pipe surfaces to evaluate pit characteristics such as bit depth, area, volume, diameters, density, and roundness. The investigation showed that the analysis of 2D SEM images and the reconstructed 3D images was possible to characterize the geometric features of the pit. The software calculates using mathematical regressions based on the statistical parameters obtained from SEM images related to surface morphology and pitting conditions. The behavior of pits on the buried pipes can be deep, shallow, and wide forming rough surfaces. Maximum pit depth was evaluated by adding minimum depth (Z-min) and height (Z-max) in-situ condition after uniform corrosion attacked the thickness of the pipe during service time. The morphology showed that pitting corrosion shared a large contribution to the failure of pipe structures during long exposure of service time. Mountains surface analysis software was effective to detect the morphology of corroded surfaces at the micro-level and was able to measure pit depth, diameter, area, roundness, and volume in a small micrometer precision from the source of SEM image. The characteristics and features of corrosion-damaged surface indicated the morphology of pit depth, cracks, cavity, and peaks as shown in Figure 8. The major oxides present on the surfaces of the pipe are Fe_3_O_4_ and Fe_2_O_3_. The depth of the pit is increasing due to the release of metallic ions at the anode during electrochemical reaction which creates pipe surface damage. Other pit characterization topographies including maximum and minimum diameters, pitting density, the shape of pitting such as roundness were extracted from 2D pseudo color images shown in Figure 9. The image was analyzed using watershed segmentation based on the ISO 25178-2 standard. The image was fully segmented as shown in Figure 9 and filled with watershed to detect pit depth, area, volume, roundness, and diameters from a 3D image which was generated from 2D. As the image processing indicates, the value of roundness is less than one (<1) which depicts the opening shape of the pit as hemispherical as shown in Figure 10e. During the study of corrosion characterization at the surface and subsurface of iron pipes, micro-cracks were identified. This scenario happens as the depth of the pit increases. Water pressure and stresses were responsible for the occurrence of cracks. Micro-cracks begin at the subsurface of the pipe due to narrow pits penetrating the pipe surface and leads to crack formation, propagation, and growth. 

### 3.5. Analysis of Corrosion Damage Mechanisms of the Pipe Surface

Corrosion is an unwanted result obtained from the reaction between the environment and materials with time function. The pipe surface deterioration caused by soil corrosivity, water environment, buried service time, and pipe materials are very important parameters that influence buried water pipes to surface damage. As the exposure time increased, the service life of pipelines decreased. Numerous factors lead to pipe failure. Some of them are easily identified by the inspection of pipeline and its surrounding environment while others require more in-depth investigation of failure mechanisms relating to pipe material, age, manufacturing method, operating conditions, soil load, soil type, etc. are the main corrosion damage mechanisms.

In this study, the external pipe corrosion was evaluated and which was affected by the soil environmental parameters including pH, resistivity, total nitrogen, moisture, loam soil type, and bulk density whereas the internal pipe corrosion mechanisms were the low level of dissolved oxygen (80–81 ppb), conductivity (171.4–493.4 µS/cm), CaCO_3_ (77–215 ppm), total dissolved solids (TDS) (84.10–262.8 ppm), flow rate (1.5–2.5 m/s, internal pressure is 350 KPa, service load, and ClO_2_, (0–0.5 ppm). The topography of corroded surface filled with watershed segmentation which was generated from the SEM image as shown in Figure 9. It displays that the image was divided automatically by the software into several particles and labeled by the numbers with grain boundaries. This was important to characterize corrosion features as per particle level. After characterization of each particle, the results were summarized by the software itself in the form of a table and graphs as is depicted in Figure 10. 

Figure 10a–f presents summary result of corrosion characterization of image processing analysis including pit depth, maximum height from the neutral line, pit volume, pit area, maximum pit diameter and pit shape. The figures show the outputs of watershed segmentation of the entire SEM image to analyze particles, grain boundaries, and other features including pit area, depth, shape, cavity, and diameters from the 2D image. The results of image processing are represented graphically in Figure 10a–f. Figure 10a shows the threshold values of pit depth which are ranged from (40–120) µm even though both values are very small which can be considered as uniform corrosion. Figure 10b presents the result of maximum height (Z-max) which is above the mean line of surface features.

The graph shows the roughness or irregularity of corrosion-damaged surface values ranging from 40 to 70 µm. Figure 10c also presents minimum and maximum of pit area ranged from 400 to 1600 µm^2^. Additionally, Figure 10d is indicating that the volume of the pit varied from 10,000 to 40,000 µm^3^ among the particles. Similarly, Figure 10e presents the evaluation of pit shapes (roundness) per particle. As it is shown on the graph, the roundness values are less than 1 µm which means the shape of the pit was elliptical. According to image processing, corrosion characterization of pitting features such as diameter is a very important parameter to describe the pitting situation with the relation of pipe surface damage. From the ISO 25178-2 watershed segmentation method; the study detected morphology of corrosion damaged surface to characterize corrosion behaviors in-situ condition Based on the study, the maximum pit diameter was 50 µm. Based on characterization, the values of pit depth, area, and volume are 390 μm, 4000 μm^2,^ and 1600 μm^3^, respectively, obtained from 20 years old corrosion-damaged GS pipe. Similarly, from the image processing; the pit depth of DIP was 380 μm, whereas maximum pitting area and volume are 4000 μm^2^ and 200,000 μm^3^, respectively, as shown in Table 4. 

**Figure 11:**
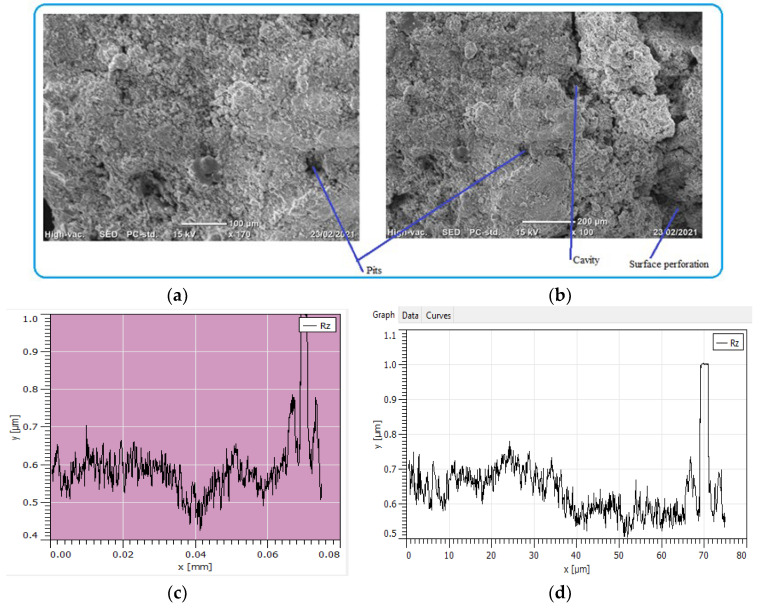
SEM images of localized and uniform corrosion morphologies of GS pipe: (**a**,**b**), and (**c**,**d**) are showing cross-sectional details of (**a**,**b**).

The process of pipe surface damage was reducing pipe thickness layer-by-layer during the electrochemical process. This is due to the formation of shallow pitting corrosion at random locations and the neighboring pitting colonies joined together to form uniform corrosion. In this case, a smaller pit depth was created on the remaining surface of the pipe. For example, the pit depth up to 400 μm can be categorized as uniform corrosion. On the other hand, localized pitting corrosion created a cavity and perforation of pipe surface while the pits grow with the function of time and environmental factors Figure 11. As the pit depth increases, the pit can hold deposits and chloride. With the presence of chloride ions, the rate of corrosion is more accelerated than the accumulation created a leakage. The magnification of images was ranged from 100× (Figure 11a) to 170× (Figure 11b) for the internal pipe wall of GS pipe.

Figure 11c presents the cross-sectional details of the corrosion-damaged pipe surface. Maximum pit depth was found at the center of the surface; similarly, a more damaged surface with pit and crack was found at the left age of the sample (Figure 11d). As compared to the two figures of cross-sections, Figure 11d has more pit depth and crack propagates than Figure 11c. 

**Figure 12:**
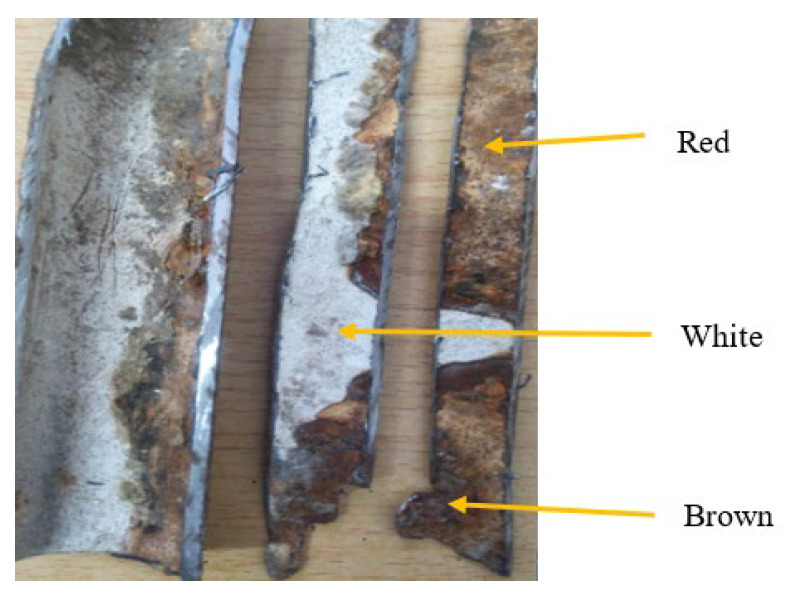
Photographs of the typical corroded pipe surface.

A corrosion pit was caused by scratch, localized surface defect, environmental corrosion, and variations of alloy compositions. Corrosion products have different colors such as white, red, and brown Figure 12. Corrosion product that had white color was ZnO and the red color represented as Fe_3_O_4_ while brown color was identified as Fe_2_O_3_. The white color ZnO was observed at some random points on the surface of the pipes and the geometry of corrosion had different sizes, shapes, and depths of localized corrosion. 

**Figure 13:**
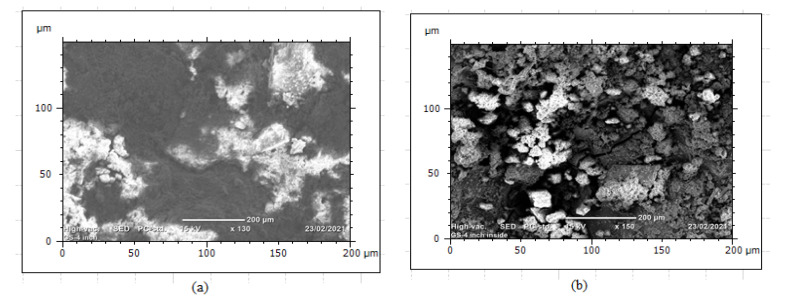
SEM image of corrosion damaged GS pipe: (**a**) external corrosion and (**b**) internal corrosion.

The surface irregularities observed on both internal and external surfaces were a result of soil and water effect during long-run service years. After the protecting of zinc coating has oxidized, the steel surface begins to form corrosion scales around the pipe and localized corrosion damage continued to the subsurface of the pipes even further attacked the whole pipe thickness. The internal pipe corrosion was a result of hydraulic operating conditions that influence the formation of erosion–corrosion and cracks due to water pressure and the effect of stagnant water during a steady time. Figure 13 shows that the morphology of internal corrosion was different from external corrosion due to inherent variations of environmental corrosion mechanisms. The study of corrosion-damaged pipe surface characterization helps to investigate water quality from the rate of corrosion. This is because the water is directly in contact with the pipe surface. Thus, it requires regular inspection and testing of water. In this case, when internal pipe surface damages due to corrosion and operating conditions, the release of pipe material affects the water quality. The water distribution owners need to test the water after treatment to evaluate the corrosion rate. It was also very important to the industries to evaluate corrosion behavior on the iron material at any stage to control the risks of corrosion. 

In this study, as compared with corrosion resistance between GS and DIP, both pipes have different corrosion rates. The DIP pipe had more corrosion resistance capacity than GS due to variations of parameters including the thickness of the pipe, coating type, pipe compositions, and buried depth. For example, the thickness of DIP and GS pipes were 8 mm and 5 mm, respectively. Similarly, the buried depth of the DIP water main was 120–150 cm whereas the GS pipe is buried at 80 cm depth for 100 mm diameter. Due to mechanical properties and coating type of bituminous coating, DIP pipe is more corrosion-resistant than GS pipe. From a variety of corrosion characterizing methods, the results show that pitting corrosion is the main pipe failure problem of water pipes. The external pipe surface of the DIP pipe is affected by uniform corrosion. Similarly, the internal pipe wall was damaged by a corrosion pit and crack. The subsurface was affected by the crack due to water pressure and narrow pits. In general, pipe surface damage was a complex problem due to the combined effect of electrochemical reactions and stresses applied to the pipe. 

## 4. Conclusions

This study explored characterizations of corrosion morphologies at the surface and subsurface of corrosion-damaged iron-based water pipes. The proposed method of corrosion characterized using Mountains 8.2 digital image surface analysis software was used to analyze corrosion damaged surface at any stage up to particle level. The acceptability of corrosion defects was evaluated using image processing based on the ISO 25178-2 standard, microstructure analysis, and corrosion morphology characteristics. The rate of corrosion was more at the subsurface of the pipes than the outer surface as a result of pit depth growth. The SEM analysis confirmed that micro-cracks were observed at the subsurface due to water pressure and pitting depth. On the other hand, microstructural evaluation using OM showed the grain size and boundaries of the structure were observed as uniform distribution of alloying elements. From the various characterization techniques, the rate of corrosion deterioration process was categorized as: (1) pipe coating corrosion damage; (2) external pipe surface weakening; and (3) subsurface corrosion degradation and failure of pipe structures. 

This study provided a basic understanding of water pipe corrosion damage processed at the surface and subsurface of the pipes to understand the causes of water quality deterioration and structural failure. The outcome of this study will be important for the water distribution system, pipe manufacturers, and industries to control corrosion at an early stage.

## Figures and Tables

**Table 1:** Elemental compositions of galvanized steel pipe.

Elements by wt%										
C	Mn	Si	Ni	P	Mo	Cr	S	V	Al	Fe
1.89	0.41	0.09	0.06	0.01	0.01	0.10	0.01	0.09	0.05	Bal.

**Table 2:** The material composition of ductile iron pipe in wt%.

Elements												
Pipe	C	Mn	P	S	Si	Ni.	Cr	Mo	Cu	Ti	Mg	Fe
DIP	3.600	0.340	0.090	0.032	2.250	0.060	0.070	0.000	0.080	0.14	0.008	Balance

**Table 3:** Soil physicochemical parameters.

Soil Parameters	Lab Test Value
Moisture content (%)	23.7–37.5%
pH	6.98–7.04
Electrical conductivity (ds/m)	0.105–0.313
Total nitrogen (%)	0.06
Soil texture class	Loam
Soil chloride concentration (mg/Kg)	500–1000

**Table 4:** A comparison of pitting features between GS and DIP pipes.

Pipe Type	Pit Depth (µm)	Diameter (µm)	Area (µm^2^)	Volume (µm^3^)	Roundness
GS	310–390	20–50	4000	16,000	0.7
DIP	290–380	28–60	4000	200,000	0.8

## Data Availability

Not applicable.
